# A Rare Surgical Case of a Posterior Rectus Sheath Hernia Causing Small Bowel Obstruction

**DOI:** 10.7759/cureus.96248

**Published:** 2025-11-06

**Authors:** Konstantina Kostara, Ahmer Sultan, YaQun Zhou, Benetta Miller, Zbigniew Moszczynski

**Affiliations:** 1 Surgery, CarePoint Health, Bayonne, USA

**Keywords:** arcuate line, incisional hernia, intraparietal hernia, posterior rectus sheath, rectus sheath, retrorectus hernia, ventral hernia

## Abstract

Intraparietal hernias are a rare type of ventral hernia in which the hernia sac is located between abdominal wall layers. A posterior rectus sheath hernia, as described in this case, is a type of intraparietal hernia in which the hernia sac is located between the defect in the posterior sheath and an intact anterior sheath. These hernias can pose a diagnostic challenge and carry a higher risk of incarceration and strangulation, given the intraparietal location and often small size of the defect. Posterior rectus sheath hernias are rare, with only a few reported in the literature. In this case report, we describe a posterior rectus sheath hernia causing small bowel obstruction in a 41-year-old female patient with a surgical history of a cesarean section six weeks prior to presentation.

## Introduction

Approximately five million people in the United States suffer from an abdominal wall hernia; of these, one-third consist of ventral hernias. Of the ventral hernias that are repaired, one-third are incisional and two-thirds are primary ventral hernias [1]; 10%-15% of all patients who have previously undergone abdominal surgery will develop an incisional ventral hernia [1]. These hernias can develop after any type of incision; however, midline incisions have the highest incidence [2]. Classically, incisional hernias are a result of the breakdown of the fascial closure; this can be a result of both patient and technical factors. Patient factors include advanced age, obesity, smoking, malnutrition, immunosuppression, and connective tissue disorders. Technical factors include surgical site infection and suboptimal fascial closure, which result in excess tension and do not achieve a suture-to-wound length ratio of at least 4:1 [3]. 

Intraparietal hernias are a rare type of ventral hernia that involves the hernia sac located between abdominal wall layers [4]. A posterior rectus sheath hernia, as described in this case, is a type of intraparietal hernia in which the hernia sac is located between the defect in the posterior sheath and an intact anterior sheath. Posterior rectus sheath hernias are rare, with only 10 reported in the literature, the first of which was in 1937 [5]. In this case report, we present the unique case of a 40-year-old woman who presented with high-grade small bowel obstruction from a retrorectus incisional hernia. In this unique case, the anterior fascia was intact, and the defect was found to be through the posterior sheath and rectus muscle. Recent reports have proposed a dedicated cesarean section hernia classification that organizes post-cesarean abdominal wall hernias into five types based on defect localization and the herniation pathway.

## Case presentation

**Figure 1:**
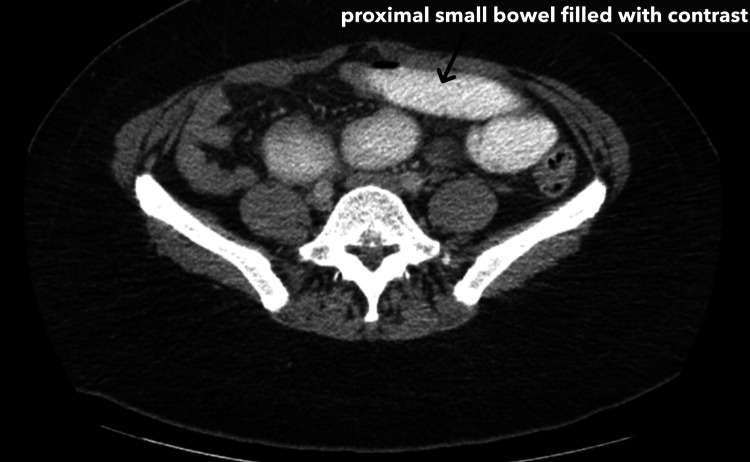
CT scan findings of the dilated, contrast-filled small bowel proximal to the hernia site The axial slide of the CT with oral contrast shows a dilated, contrast-filled portion of the small bowel, which is proximal to the site of the hernia. The proximal dilation indicates obstruction of the small bowel at the site of the hernia.

**Figure 2:**
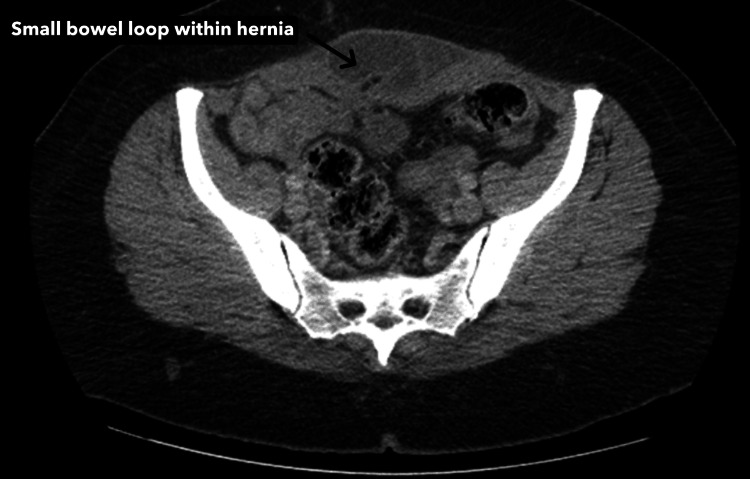
CT scan findings of the small bowel loop within hernia; no contrast seen within the incarcerated loop. The axial slide of the CT with oral contrast shows an incarcerated loop of the small bowel within the hernia. The lack of oral contrast within the incarcerated loop is indicative of obstruction at the site of the hernia.

**Figure 3:**
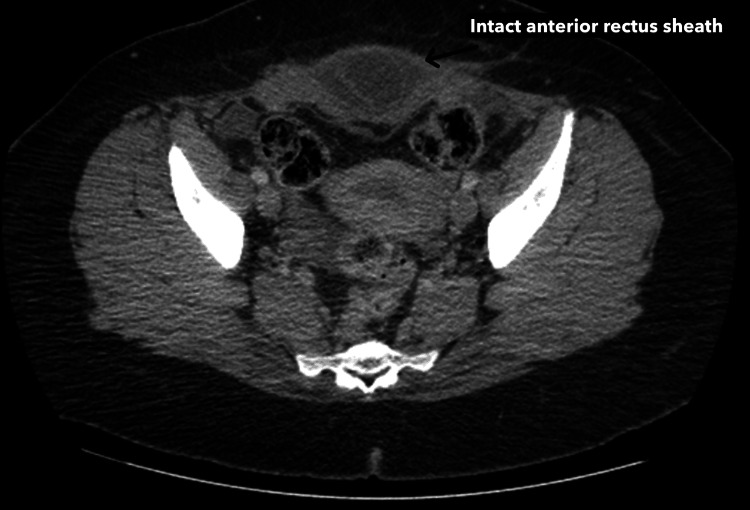
CT scan findings of an incarcerated small bowel loop with intact anterior rectus sheath This axial slide of the CT with oral contrast shows an incarcerated loop of small bowel within the hernia, with an intact anterior rectus sheath evident. The finding of the small bowel between the posterior and anterior rectus sheaths is indicative of an intraparietal hernia.

**Figure 4:**
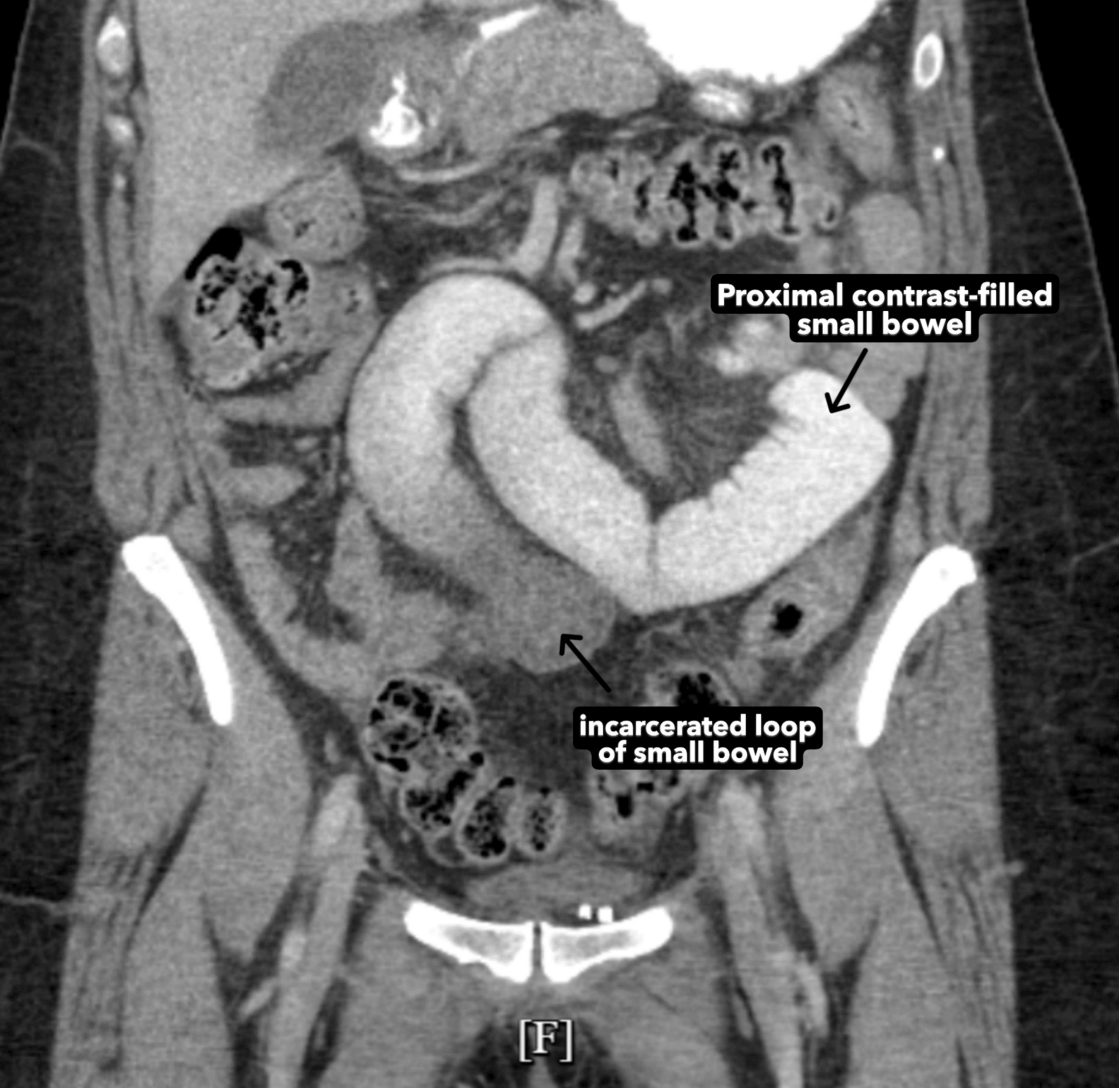
CT scan findings of an incarcerated bowel loop in the lower midline abdomen (coronal view) This coronal slide of the CT with oral contrast shows a dilated, contrast-filled portion of the small bowel that is proximal to the site of the hernia. The proximal dilation indicates obstruction of the small bowel at the site of the hernia. The lack of oral contrast within the incarcerated loop is indicative of obstruction at the site of the hernia.

**Figure 5:**
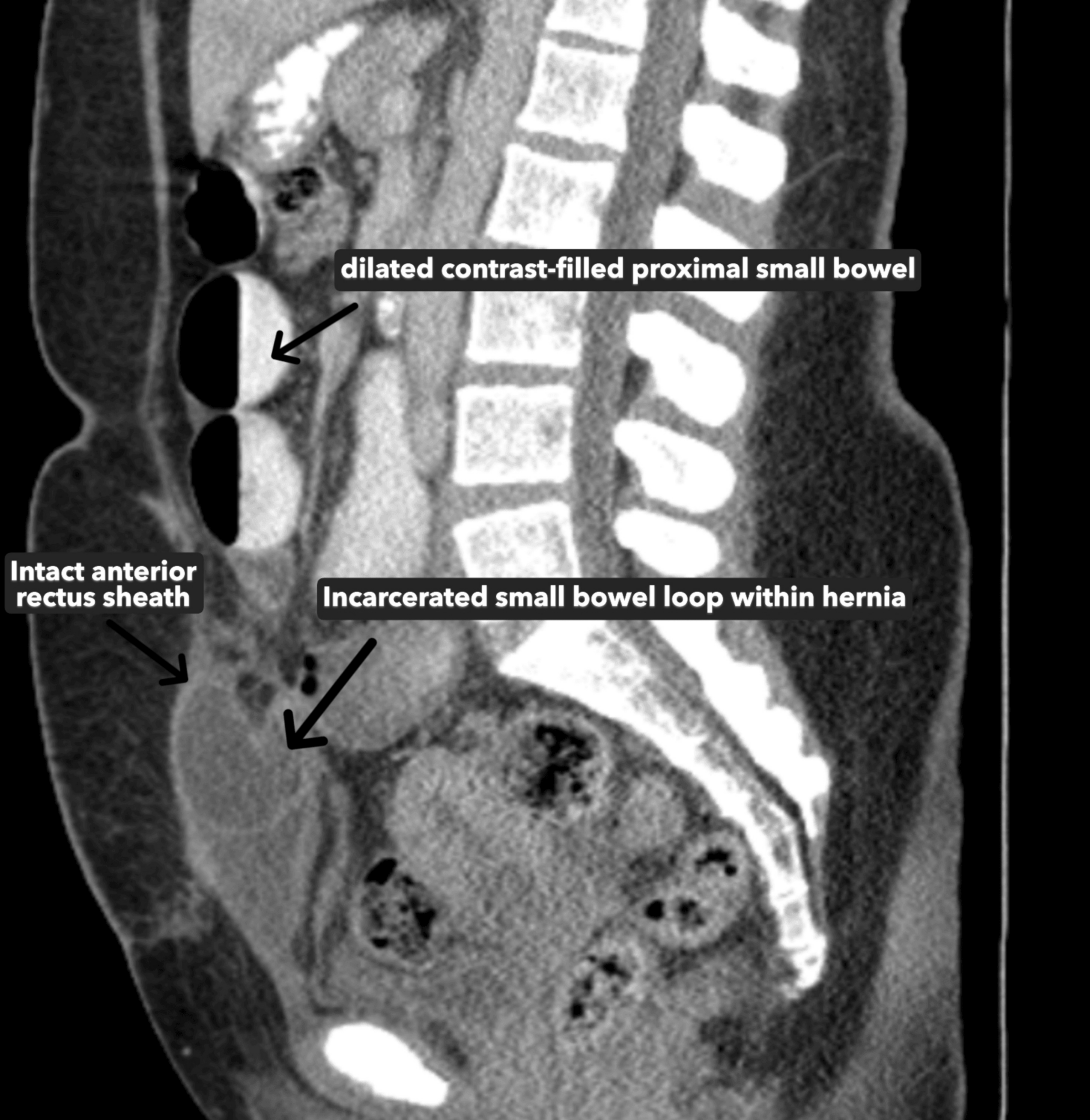
CT scan findings of an incarcerated small bowel loop within the anterior abdominal wall hernia with intact anterior rectus sheath (sagittal view) This sagittal slide of the CT with oral contrast shows a dilated, contrast-filled portion of the small bowel, which is proximal to the site of the hernia. The proximal dilation indicates obstruction of the small bowel at the site of the hernia. The lack of oral contrast within the incarcerated loop is indicative of obstruction at the site of the hernia. This image also shows an incarcerated loop of small bowel within the hernia, with an intact anterior rectus sheath evident. The finding of small bowel between the posterior and anterior rectus sheaths is indicative of an intraparietal hernia.

This is the case of a 41-year-old female patient who presented six weeks status post a cesarean section, via a Pfannenstiel incision at the skin and a vertical midline incision at the level of the fascia, with one day of intractable vomiting, abdominal pain, and obstipation. Her vitals were stable, and laboratory results, including serum lactate, were unremarkable (Table 1). Other than being six weeks post-cesarean, the patient had no other surgical or medical history, and she denied any similar prior episodes. On physical exam, her prior surgical incision was well-healed without erythema or tenderness. The lower abdomen was tender, with a small hernia palpated above the transverse scar, containing bowel. Attempts to reduce the hernia contents were ineffective. A CT of the abdomen and pelvis showed a high-grade small bowel obstruction secondary to an incarcerated bowel loop in the lower midline abdomen (Figures 1-5). 

**Table 1:** The patient's lab values upon presentation with reference values for comparison This table aims to showcase the relatively benign laboratory findings of this patient, who presented with small bowel obstruction secondary to an incarcerated bowel within a posterior rectus sheath hernia.

Test	Lab Value (K/uL)	Reference Range (K/uL)
White blood cell count	9.6	3.8 - 11.8
Hemoglobin	13.8	10.9 - 14.3
Hematocrit	40.2	31.2 - 41.9
Platelets	266	179 - 408
Neutrophils %	83.2	42.7 - 76.8
Blood urea nitrogen	19	Jul-17
Creatnine	0.8	0.7 - 1.2
Lactate	0.8	0.7 - 2.1

Given the image findings and inability to reduce the hernia at bedside, the patient was taken to the operating room on the day of presentation. Given the small bowel distention and likely need for resection and anastomosis, the preference of the operating surgeon was to proceed with laparotomy without an initial attempt at a laparoscopic approach. A midline infraumbilical incision was made over the palpable defect. The skin incision was extended down until the anterior fascia was encountered, which was found to be intact. The anterior rectus fascia was incised, and a portion of small bowel was encountered protruding through the rectus muscle, originating through a defect in the posterior rectus sheath. There was surrounding serosanguinous fluid, but the bowel itself was found to be viable and intact. The bowel was freed and found to be well-perfused and peristaltic. The 7 mm hernia defect was closed primarily with Prolene suture (Ethicon, part of Johnson & Johnson MedTech, Raritan, NJ). Per surgeon preference, a 3x6 onlay Prolene mesh (Ethicon) was placed over the primary closure and secured with an interrupted 3-0 polydioxanone suture (PDS) suture circumferentially. The anterior sheath was then reapproximated with looped 0-PDS in a running fashion. The surgical site was copiously irrigated, and the skin was approximated with staples. The patient remained stable for the duration of the operation and was admitted to the surgical floor postoperatively. She resumed bowel function on postoperative day 1 and was discharged home in stable condition.

## Discussion

Posterior rectus sheath hernias are a very uncommon subtype of interparietal hernia, in which abdominal contents pass through a defect in the posterior sheath and lie between the rectus muscle and an intact anterior sheath. Since the first description in 1937, only a small handful of cases have been documented in the literature [4,5,6]. Because of this rarity, the diagnosis can be easily overlooked, particularly in the early postoperative setting. Within the Zanellato ceseaean section hernia classification, our patient fits an infra-umbilical midline intraparietal pattern: a posterior rectus sheath defect with bowel lying between the rectus muscle and an intact anterior sheath [7]. Referencing the scheme standardizes reporting and helps distinguish these lesions from more common primary ventral or classic incisional defects [7].

Anatomically, the posterior rectus sheath is strongest above the arcuate line, where it contains contributions from the internal oblique and transversus abdominis aponeuroses. Below that line, only the transversalis fascia and peritoneum remain posterior to the rectus muscle, creating a naturally weaker segment [8,9]. This structural vulnerability may predispose to herniation, especially when combined with prior incisions, trauma, or periods of elevated intra-abdominal pressure. In our patient’s case, a recent cesarean section was the most likely precipitating factor, with the postpartum increase in abdominal wall strain possibly contributing to fascial disruption [9,10]. Regarding this particular case, it is important to note that while the traditional Pfannenstiel incision is a curved transverse incision created two fingerbreadths above the pubic symphysis, i.e., below the arcuate line, the subcutaneous dissection and fascial incision are done in a vertical fashion at the midline and often extend above the arcuate line. In this case, it is suspected that upon fascial closure, the posterior rectus sheath, below the umbilicus but above the arcuate line, was not approximated, thereby leaving a defect for bowel to herniate through. 

The presentation of posterior rectus sheath hernias is not uniform. Some patients are found incidentally on imaging for unrelated complaints [9], while others develop intermittent pain or an abdominal wall bulge that can progress to complete small bowel obstruction [6,9]. The intact anterior sheath can make physical diagnosis challenging. For this reason, computed tomography has become the key diagnostic tool, as it allows clear visualization of bowel loops positioned between the rectus muscle and posterior sheath and confirmation that the anterior fascia is preserved [4,6]. These CT features are consistent with the descriptors used in the proposed C-section hernia classification [7]. In our case, CT findings prompted urgent exploration, which confirmed the imaging diagnosis. 

Definitive management is surgical. Small, well-defined defects can be closed primarily, but reinforcement with mesh is often recommended for larger defects or attenuated tissue to reduce the chance of recurrence [4,9]. Both open and laparoscopic approaches have been reported; the latter may offer less postoperative pain and quicker recovery when feasible, though open repair remains preferred when rapid access to potentially compromised bowel is required [9,11]. Outcomes in published cases are generally favorable if intervention occurs before strangulation or ischemia develops [4,5,9].

The present case is unusual in its timing, only six weeks after cesarean section, and in the absence of bowel compromise despite a high-grade obstruction. By adding this example to the literature, we hope to increase awareness of posterior rectus sheath hernias and encourage consideration of this diagnosis in postoperative patients with small bowel obstruction and an intact anterior abdominal wall on imaging.

## Conclusions

Posterior rectus sheath hernias remain an exceptionally rare but important cause of small bowel obstruction. They are defined by herniation between the rectus muscle and the posterior sheath, with preservation of the anterior fascia. CT is the most useful tool for diagnosis and prompt surgical repair, with either primary or mesh-reinforced, which yields excellent results. This case demonstrates that even early after obstetric surgery, a posterior rectus sheath hernia can occur, and clinicians should keep it in mind when evaluating postoperative abdominal wall masses or unexplained obstruction. Increased awareness among surgeons may facilitate earlier recognition and prevent bowel ischemia and patient morbidity.
